# Glucocorticoids induce differentiation and chemoresistance in ovarian cancer by promoting ROR1-mediated stemness

**DOI:** 10.1038/s41419-020-03009-4

**Published:** 2020-09-23

**Authors:** Hanna Karvonen, Mariliina Arjama, Laura Kaleva, Wilhelmiina Niininen, Harlan Barker, Riitta Koivisto-Korander, Johanna Tapper, Päivi Pakarinen, Heini Lassus, Mikko Loukovaara, Ralf Bützow, Olli Kallioniemi, Astrid Murumägi, Daniela Ungureanu

**Affiliations:** 1 Faculty of Medicine and Health Technology, Tampere University, 33014 Tampere, Finland; 2 Institute for Molecular Medicine Finland, FIMM, University of Helsinki, 00290 Helsinki, Finland; 3 Fimlab Ltd., Tampere University Hospital, 33520 Tampere, Finland; 4 Department of Obstetrics and Gynecology, University of Helsinki, Helsinki University Hospital, Helsinki, Finland; 5 Department of Pathology, University of Helsinki and HUSLAB, Helsinki University Hospital, PO Box 400, 00290 Helsinki, Finland; 6 Science for Life Laboratory, Department of Oncology and Pathology, Karolinska Institutet, 171 65 Solna, Sweden; 7 Applied Tumor Genomics Research Program, Faculty of Medicine, University of Helsinki, FI-00014 Helsinki, Finland

**Keywords:** Cancer stem cells, Oncogenes, Cancer models

## Abstract

Glucocorticoids are routinely used in the clinic as anti-inflammatory and immunosuppressive agents as well as adjuvants during cancer treatment to mitigate the undesirable side effects of chemotherapy. However, recent studies have indicated that glucocorticoids may negatively impact the efficacy of chemotherapy by promoting tumor cell survival, heterogeneity, and metastasis. Here, we show that dexamethasone induces upregulation of ROR1 expression in ovarian cancer (OC), including platinum-resistant OC. Increased ROR1 expression resulted in elevated RhoA, YAP/TAZ, and BMI-1 levels in a panel of OC cell lines as well as primary ovarian cancer patient-derived cells, underlining the translational relevance of our studies. Importantly, dexamethasone induced differentiation of OC patient-derived cells ex vivo according to their molecular subtype and the phenotypic expression of cell differentiation markers. High-throughput drug testing with 528 emerging and clinical oncology compounds of OC cell lines and patient-derived cells revealed that dexamethasone treatment increased the sensitivity to several AKT/PI3K targeted kinase inhibitors, while significantly decreasing the efficacy of chemotherapeutics such as taxanes, as well as anti-apoptotic compounds such as SMAC mimetics. On the other hand, targeting ROR1 expression increased the efficacy of taxane drugs and SMAC mimetics, suggesting new combinatorial targeted treatments for patients with OC.

## Introduction

Epithelial ovarian cancers (OCs), of which 70–80% are high-grade serous ovarian cancer (HGSOC), are the leading causes of gynecological cancer death in developed countries^1^. The standard OC treatment based on tumor debulking followed by platinum and taxane-based chemotherapy leads to responses in 60–70% of cases^2^. However, relapse due to acquired resistance is very common and the five-year survival of HGSOC cases is less than 40%^3^. Another subtype of epithelial OCs is low-grade serous ovarian cancer (LGSOC), which is characterized by slow progression as well as resistance to conventional chemotherapy^4^. Therefore, a key therapeutic goal in OC treatment is to optimize chemotherapy efficacy in order to eliminate residual tumor cells.

Patients with advanced cancer often suffer major complications, such as the brain, spine, and other edemas, or severe systemic side effects of chemotherapy. These and other complications are often mitigated with dexamethasone (DEX), a synthetic glucocorticoid that activates the same nuclear glucocorticoid receptor (GR) as natural stress hormones, such as cortisol and corticosterone^5,6^. However, glucocorticoids have been shown to directly impact OC tumor development by decreasing the efficacy of chemotherapy through inhibition of apoptosis, indicating that DEX could impair the effectiveness of OC chemotherapy^7,8^. Interestingly, recent transcriptomic and proteomic analysis of breast cancer models showed that DEX-mediated GR signaling activation promoted metastasis by upregulating the non-canonical Wnt pathway highlighted by ROR1 (receptor tyrosine kinase-like orphan receptor) expression while decreasing the efficacy of paclitaxel^9^. These findings point toward the existence of a positive feedback loop between GR signaling activation and upregulation of ROR1 expression in metastatic breast cancer cells, prompting us to investigate this signaling loop in OC models.

The ROR family of proteins belongs to the non-canonical Wnt pathway and is comprised of two receptors, ROR1 and ROR2 that can bind Wnt5a ligand via their extracellular domain^10^. In OC, both ROR1 and ROR2 are important for cell growth, migration, and invasion^11^, while high levels of ROR2 correlated with the development of platinum resistance^12^. Furthermore, ROR1-positive OC cells have stemness properties, as demonstrated by high levels of ALDH1 or cell surface expression of cancer stem cell (CSC) markers such as CD133 and CD44^13^. Indeed, ROR1 expression is also a marker for the shorter overall survival of OC patients^14^.

In this study, we demonstrate that DEX treatment upregulates ROR1 expression in OC models (cell lines and patient-derived primary cells—PDCs) including platinum-resistant cells, cultured in 2D or 3D-spheroid conditions. We found that the DEX-mediated increase of ROR1 levels correlated with the upregulation of RhoA GTPase, Hippo signaling effectors YAP/TAZ as well as BMI-1 expression, resulting in stemness phenotype and differentiation of OC tumor cells, including platinum-resistant samples. Furthermore, high-throughput drug sensitivity and resistance testing (DSRT, 528 compounds) identified that DEX enhanced the efficacy for targeted AKT/PI3K kinase inhibitors and decreased the cytotoxic effect of conventional chemotherapeutics, taxanes, and SMAC mimetics. On the other hand, shRNA targeting of ROR1 expression increased the efficacy of SMAC mimetics and taxanes. Collectively, our data provide new evidence for the effect of glucocorticoids on OC disease biology as well as on drug responses. The impact of DEX on the OC cells drug responsiveness to clinically relevant drugs could have implications on clinical disease management. Targeting ROR1 expression may counter this effect and provide therapeutic advances.

## Materials and methods

### Reagents

Cisplatin, paclitaxel, NVP-LCL161, birinapant, and AT-406 were obtained from Selleckchem (Houston, TX, USA). Doxycycline, verteporfin, and water-soluble form of dexamethasone were from Sigma-Aldrich (Merck, Darmstadt, Germany) and recombinant Wnt5a from Bio-Techne (Minneapolis, MN, USA). Experimental methods and related details are summarized in Supplementary Methods.

## Results

### Wnt5a-ROR pathway is expressed in platinum-resistant OC models

**Fig. 1:**
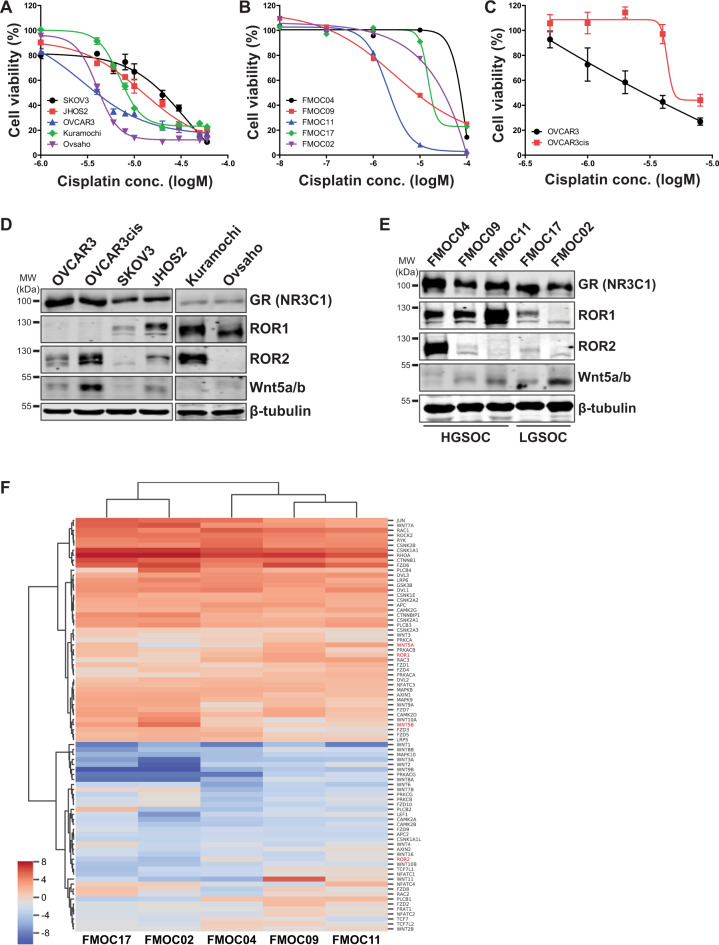
Analysis of cisplatin sensitivity and the expression of Wnt5a, ROR1, ROR2, and NR3C1 in OC cell lines and PDCs. **a**, **b** The sensitivity of OC cell lines **a** OVCAR3, JHOS2, SKOV3, Kuramochi, Ovsaho, and patient-derived primary cells (PDCs) **b** to cisplatin was tested with cell viability assay after 72 h incubation with various cisplatin concentrations as indicated. The bars represent mean ± SD. **c** The sensitivity of OVCAR3 and OVCAR3cis to cisplatin was measured by cell viability assay after 72 h incubation with various concentrations of cisplatin. The bars represent mean ± SD. OVCAR3cis showed high resistance to cisplatin cytotoxicity. **d**, **e** Western blot analysis of Wnt5a, ROR1, ROR2, and NR3C1 expression in OC cell lines (**d**) and PDC (**e**) cell lysates. β-tubulin was used as a loading control. **f** Hierarchical clustering of expression of KEGG defined Wnt-pathway genes^47^. Values are presented as log2 transformed transcripts per kilobase million (TPM) from RNA-Seq from five PDCs; (blue = low; red = high). HGSOC high-grade serous ovarian cancer, LGSOC low-grade serous ovarian cancer.

Platinum resistance is a major problem associated with OC therapy outcome, therefore we examined cisplatin sensitivity in five representatives OC cell lines and five PDCs (three HGSOC and two LGSOC PDCs). Table 1 provides the diagnosis and clinical characteristics of PDCs used in this study. Two PDCs (HGSOC/FMOC04 and LGSOC/FMOC02) were established from patients with chemoresistant, recurrent disease. The PDCs established from ascites and tumor tissue samples represent clinically representative models for predicting drug treatment efficacy, as they may recapitulate sensitivity and resistance patterns and mechanisms in patients^15^. We observed various sensitivities for cisplatin in OC cell lines and PDCs (Fig. 1a, b). Since OVCAR3 cells were more sensitive to cisplatin, we developed a cisplatin-resistant OVCAR3 variant (OVCAR3cis, Fig. 1c) to uncover changes in intracellular signaling associated with cisplatin resistance. Western blot analysis of the non-canonical Wnt pathway (Fig. 1d, e) revealed increased Wnt5a-ROR2 expression in OVCAR3cis compared to OVCAR3 parental cells. SKOV3, JHOS2, and Kuramochi cell lines that showed high inherent primary resistance to cisplatin showed a high expression of ROR1. Hierarchical clustering showed that based on the expression of Wnt-pathway genes, the HGSOC PDCs (1, 2, and 3) clustered together while, likewise, expression values for LGSOC PDCs were most similar to each other. In addition, across all samples, Wnt-pathway genes related to non-canonical Wnt signaling and planar cell polarity (PCP) pathways had higher levels of expression than those more associated with Frizzled binding (Fig. 1f). Furthermore, all HGSOC PDCs showed high ROR1 levels (Fig. 1e, f), whereas ROR2 expression was detected most strongly in FMOC04 (derived from a chemoresistant patient), corroborating a previous gene expression analysis showing high expression of ROR1 in HGSOC samples compared to other OC subtypes^13^. Moderate expression of Wnt5a could be seen in some PDCs, notably FMOC02. All OC cell lines and PDCs showed a high expression of NR3C1 (GR), indicative of active glucocorticoid signaling.

**Table 1:** Diagnosis and clinical characteristics of ovarian cancer PDCs.

Patient ID	Histological subtype	Stage	Disease stage	Sample type
FMOC04	HGSOC	IVA	Recurrent (peritoneal metastases)	Ascites
FMOC09	HGSOC	IIIC	Primary	Tissue
FMOC11	HGSOC	IVA	Primary	Tissue
FMOC17	LGSOC	IVA	Primary	Tissue
FM0C02	LGSOC	IIIC	Recurrent (peritoneal metastases)	Ascites

### Glucocorticoids upregulate of Wnt5a-ROR signaling in OC models

**Fig. 2:**
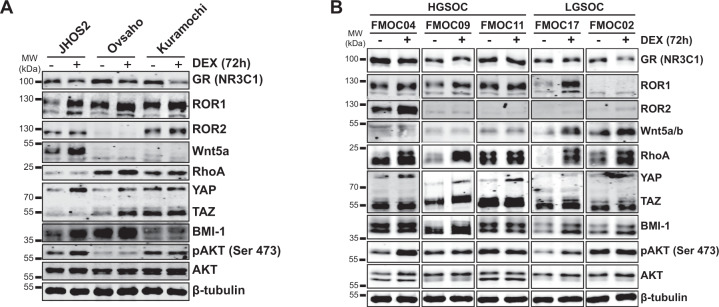
DEX treatment enhances the expression of ROR1 and its downstream signaling in OC models. **a** OC cell lines JHOS2, Kuramochi, and Ovsaho were left untreated or treated with 100 nM DEX for 72 h, followed by western blot analysis of respective protein levels as indicated. β-tubulin was used as a loading control. **b** OC PDCs were cultured ex vivo and untreated or treated with 100 nM DEX for 72 h, followed by western blot analysis of respective protein levels as in **a**. β-tubulin was used as a loading control. A representative of three technical replicates is shown for each panel.

Next, we sought to investigate whether glucocorticoids could modulate ROR1 expression in OC as recently demonstrated in breast cancer preclinical models^9^. We treated OC cell lines for 72 h with 100 nM DEX, a concentration corresponding to plasma levels of DEX when administered to cancer patients^6^, followed by western blot and flow cytometry analysis. Our results show that DEX treatment enhanced ROR1 expression in JHOS2, Ovsaho, and Kuramochi cells (Fig. 2a and Supplementary Fig. S1B). We also observed a DEX-mediated increase in downstream ROR1 signaling mediators such as RhoA GTPase, Hippo effectors YAP/TAZ, and polycomb ring-finger oncogene BMI-1 protein levels, with variation in every cell line. Both YAP/TAZ and BMI-1 are regulators of self-renewal, differentiation, and tumor initiation of CSCs, indicating that glucocorticoids could induce ROR1-associated stemness phenotype in OC cells^10,16,17^. Moreover, a marked increase in BMI-1 and pAKT levels were detected in DEX-treated OVCAR3 cell lysates (Supplementary Fig. S1C), despite the lack of changes in ROR1/ROR2 levels. DEX-mediated activation of pAKT was previously observed in some OC cell lines^18^.

Furthermore, we addressed the effect of glucocorticoids on the ROR1 level in cultured PDCs ex vivo (Fig. 2b). As expected, ROR1 and its downstream effectors RhoA, YAP/TAZ, and BMI-1 levels were enhanced by DEX treatment in FMOC04, FMOC09, FMOC11, and FMOC17, whereas a modest increase in DEX-mediated Wnt5a levels was detected in FMOC17 and FMOC02. ROR2 levels were also upregulated by DEX treatment in FMOC04, suggesting that both ROR receptors are susceptible to DEX-mediated expression modulation in PDCs. Elevated levels of pAKT were also detected in DEX-treated FMOC04 and FMOC17 compared to untreated samples. Interestingly, we observed a modest increase in Wnt5a and BMI-1 levels following DEX treatment of FMOC02 lacking ROR1 or ROR2 expression, but no DEX-mediated changes in YAP/TAZ, RhoA, or pAKT levels.

### Wnt5a-ROR1 signaling directly modulates YAP/TAZ expression in OC cells

**Fig. 3:**
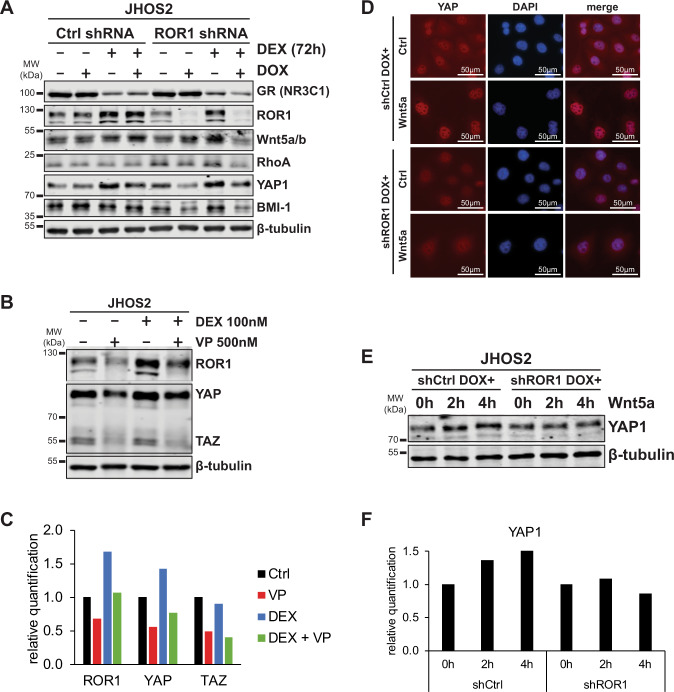
ROR1 expression associates with YAP/TAZ and BMI-1 activation in OC cells. **a** JHOS2 cells stable transfected with doxycycline-inducible Ctrl or ROR1 shRNA were untreated or treated with 100 ng/ml doxycycline for 48 h followed by 100 nM DEX treatment for an additional 72 h as indicated. Western blot analysis of total cell lysates was carried out for respective protein levels as indicated. β-tubulin was used as a loading control. **b** JHOS2 cells were untreated or treated with 100 nM DEX for 48 h followed by 500 nM verteporfin (VP) treatment for an additional 24 h as indicated. Western blot analysis of total cell lysates was performed for YAP/TAZ and ROR1 levels, while β-tubulin was used as a loading control. **c** Protein quantification from **b**. **d**, **e** DOX-treated JHOS2 cells stable transfected with Ctrl or ROR1 shRNA and treated with DOX in the presence or absence of Wnt5a stimulation for Immunofluorescence staining of YAP1 (Wnt5A 50 ng/ml for 2 h) (**d**) and western blot analysis (Wnt5a 50 ng/ml for 0/2/4 h as indicated) (**e**). **f** Quantification of YAP1 levels from **e**. Protein levels were normalized to β-tubulin and 0 h (no Wnt5a stimulation) used as a reference point (value 1) for YAP1 levels.

Previous studies have indicated the existence of a crosstalk between activation of ROR1 and YAP/TAZ signaling leading to stemness and chemoresistance^10,19^, which prompted us to investigate this feedback loop in DEX-treated OC cells. Stable expression of doxycycline-inducible shRNA targeting ROR1 in JHOS2 cells effectively downregulated ROR1 levels compared to shRNA control samples (Fig. 3a) and abolished DEX-mediated upregulation of YAP1, RhoA, and BMI-1 levels in cells lacking ROR1 expression. Downregulation of RhoA, BMI-1, and YAP/TAZ was also observed upon ROR1 knockdown in Ovsaho cells (Supplementary Fig. S3C). On the other hand, GR expression was not affected by ROR1 downregulation, suggesting that other intermediate pathway(s) could mediate this feedback loop (Fig. 3a). Moreover, inhibition of YAP/TAZ by verteporfin, a suppressor of YAP/TAZ complex, downregulated ROR1 in both, untreated and DEX-treated JHOS2 cells (Fig. 3b, c), suggesting that inhibition of YAP/TAZ negatively modulates ROR1 levels. Previous data have shown that in breast cancer cells, Wnt5a stimulation of ROR1 signaling could increase YAP/TAZ expression and nuclear localization, and this effect was ROR1-dependent^19^. Immunofluorescence (Fig. 3d) and western blot (Fig. 3e, f) analysis of JHOS2 cells treated with exogenous Wnt5a showed enhanced expression and nuclear localization of YAP1 and that this effect was ROR1-dependent, suggesting that activation of Wnt5a-ROR1 signaling directly induces YAP/TAZ upregulation in OC cells.

### Glucocorticoids modulate Wnt5a-ROR1 expression and cell differentiation in OC spheroids

**Fig. 4:**
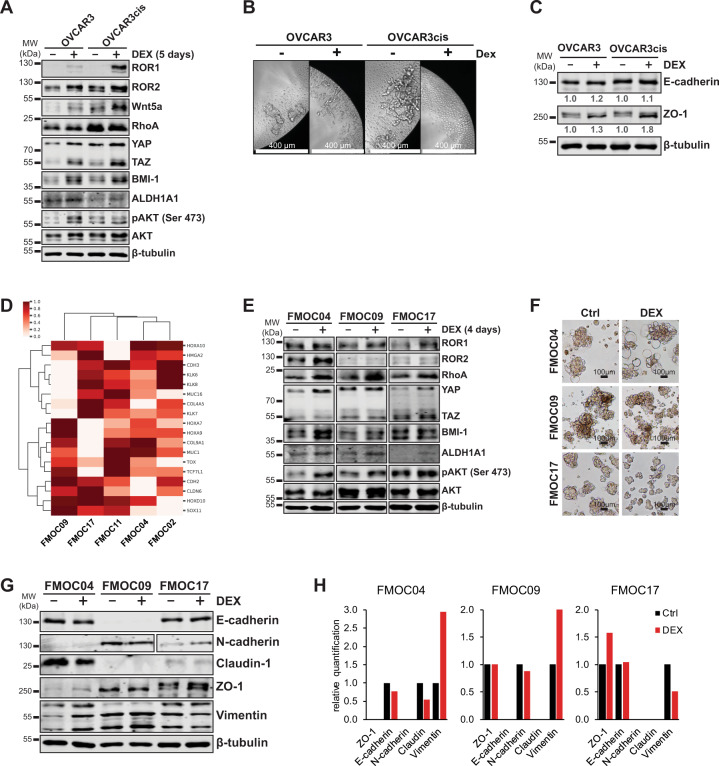
DEX treatment enhances ROR signaling (ROR1, ROR2, and Wnt5a) and stemness phenotype in OC spheroids while promoting cell differentiation. **a** Western blot analysis of OVCAR3/OVCAR3cis cells grown in spheroid conditions in the presence or absence of 100 nM DEX treatment for 5 days. β-tubulin was used as a loading control. A marked increase in ROR signaling (Wnt5a, ROR1, and ROR2 levels) and stemness markers (BMI-1, ALDH1A1, and YAP/TAZ) is observed in DEX-treated spheroids. **b** Photomicrographs of OVCAR3/OVCAR3cis grown in spheroid conditions and treated as in **a**, scale bar 400 μM. **c** Western blot analysis and quantification of E-cadherin and ZO-1 levels of OVCAR3/OVCAR3cis grown in spheroid conditions and treated as in **a**. β-tubulin was used as a loading control. Protein levels were normalized to β-tubulin and an untreated sample was used as a reference point (value 1) for quantification. **d** Hierarchical clustering of expression of mesenchymal, de-differentiated, cell type marker genes^23^ shows that FMOC09 exhibits a mesenchymal-like expression pattern; high expression of SOX11, and low expression of kallikreins. Values are presented as log2 transformed transcripts per kilobase million (TPM) from RNA-Seq from five PDCs, which have been row-normalized (zero to one). **e** Western blot analysis of PDC spheroids in the presence or absence of 100 nM DEX treatment as indicated showing upregulation of ROR1, ROR2, and its downstream YAP/TAZ and BMI-1 markers, as well as ALDH1A1 and pAKT levels in DEX-treated samples. β-tubulin was used as a loading control. **f** Photomicrographs of PDCs grown in spheroid conditions and treated as in **e**, scale bar 100 μM. **g**–**h** Western blot analysis (**g**) and protein quantification (**h**) of differentiation markers in cell lysates derived from PDCs spheroids grown as in **f**. Protein levels were normalized to β-tubulin and an untreated sample was used as a reference point (value 1) for quantification. A representative of three technical replicates is shown for each panel.

OC is known to disseminate via a direct extension of cancer cells across the peritoneal space as aggregated spheroids shedding from the primary tumor, contributing to disease progression via intraperitoneal metastatic spread^20^. Modulation of cadherins’ expression could influence OC progression via the development of peritoneal metastasis and the presence of residual tumor cells^21^. To mimic the growth of OC in vivo, we cultured cells in low-attachment conditions, to favor the spheroid formation and assessed the molecular consequences of GR activation by monitoring changes in Wnt5a-ROR1 signaling, cadherins, and cadherin-associated differentiation markers, and spheroid morphology. DEX-treated OVCAR3/OVCAR3cis cells grown in spheroid conditions displayed a marked increase in Wnt5a-ROR1/ROR2 expression, along with downstream YAP/TAZ, BMI-1, pAKT, and aldehyde dehydrogenase (ALDH1A1) levels, as shown by western blot analysis (Fig. 4a). Notably, while we did not observe a DEX-mediated increase in ROR1 expression in OVCAR3/OVCAR3cis cells grown in traditional cell culture (Supplementary Fig. S1C), we could detect ROR1 expression as well as an increase in ALDH1A1 expression in DEX-treated OVCAR3/OVCAR3cis spheroids (Fig. 4a), a strong indication of stemness phenotype in these cells. Moreover, photomicrographs showed that glucocorticoid treatment clearly impaired spheroid formation especially in OVCAR3cis cells (Fig. 4b), although cell viability was not affected (Supplementary Fig. S2). Western blot analysis showed that both OVCAR3/OVCAR3cis cells were positive for E-cadherin and ZO-1 (zonula occludens protein-1) expression (Supplementary Fig. S3A), corresponding to an epithelial-like phenotype as both proteins are involved in epithelial cell polarity^22^. We found that DEX-treated OVCAR3/OVCAR3cis spheroids showed a marked increase in ZO-1 expression compared to untreated samples (Fig. 4c), which corresponded to a loss of spheroid formation and suggests a more epithelial-like phenotype.

Next, we investigated spheroid formation in glucocorticoid-treated PDCs. Molecular profiling of differentiation markers by gene expression (Fig. 4d) and western blot (Supplementary Fig. S3B) analysis identified that FMOC09 has high expression of N-cadherin, homeobox gene SOX11 and vimentin and low kallikreins levels, corresponding to a mesenchymal-like or de-differentiated phenotype^23,24^, while other PDCs have high E-cadherin but low N-cadherin expression, indicative of an epithelial-like phenotype. Claudin expression was detected strongly in FMOC04 and FMOC11 (Supplementary Fig. S3B). Furthermore, DEX treatment of FMOC04, FMOC09, and FMOC17 grown in spheroid condition resulted in the anticipated upregulation of ROR1/ROR2 and its downstream RhoA, YAP/TAZ, BMI-1, and pAKT levels (Fig. 4e), recapitulating our finding from traditional culture conditions in Fig. 2b. DEX treatment also resulted in the upregulation of ALDH1A1 levels in PDCs spheroids, suggesting the development of the stemness phenotype in these cells. A microscopic assessment revealed that DEX-treated FMOC04 formed large spheroid-like single cells (Fig. 4f) characterized by decreased E-cadherin and claudin, but increased vimentin expression compared to untreated sample (Fig. 4g, h), indicative of a DEX-mediated mesenchymal differentiation. DEX-treated FMOC09 spheroids were morphologically identical to control (untreated), although western blot analysis showed decreased N-cadherin and increased vimentin levels while ZO-1 levels remained unchanged, suggesting an intermediate mesenchymal phenotype. However, DEX-treated FMOC17 spheroids were smaller compared to control samples and we detected elevated ZO-1 levels and a moderate decrease in vimentin, indicative of an epithelial phenotype.

### DEX augmented drug responses to targeted kinase inhibitors while impairing drug efficacy for chemotherapy and SMAC mimetics

Several studies have shown that glucocorticoids promote tumor cell survival while inhibiting chemotherapy drug responses, however, these studies were done only for a few drugs^6^. Therefore, we assessed the global changes in drug responses mediated by DEX treatment in OC by monitoring drug-sensitivity responses using a DSRT screen^25^ for a panel of 528 small molecule inhibitors (each drug in five concentrations), including established and emerging targeted cancer drugs. To obtain DEX-selective drug sensitivities, DSRT was carried out in the presence or absence of DEX treatment (100 nM) for 3 days followed by a comparison of drug-sensitivity scores (DSSs).

**Fig. 5:**
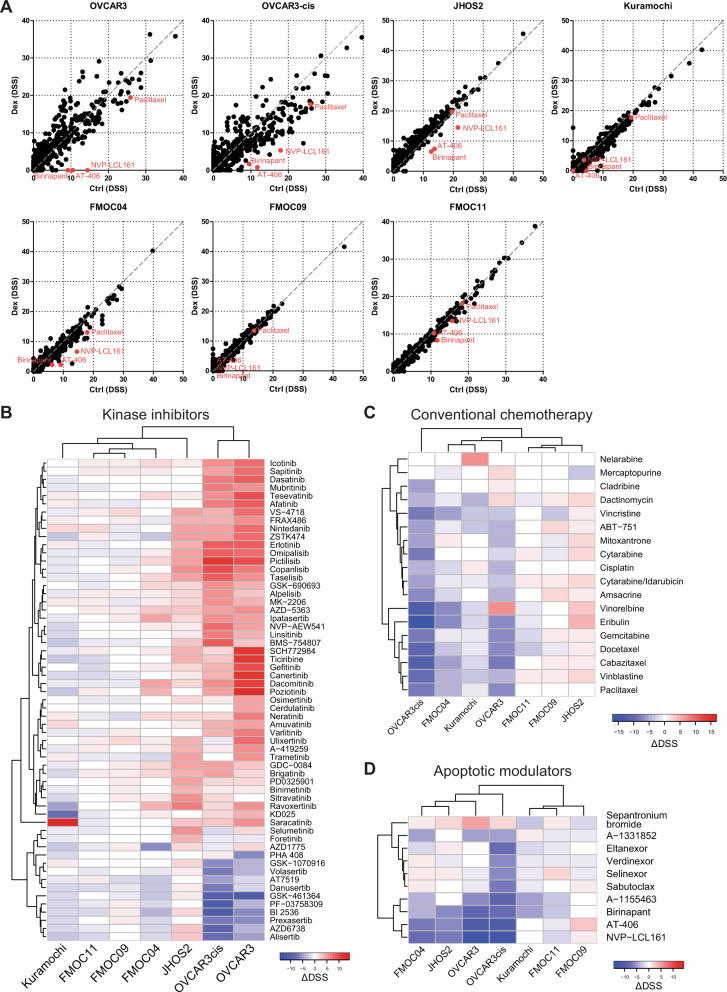
DEX treatment modulates drug sensitivities in OC cell lines and PDC. **a** Plots depicting drug-sensitivity scores (DSS) for OC cell lines and PDCs representing Ctrl (untreated) vs. DEX-treated cells. Selected SMAC mimetic drugs and paclitaxel are highlighted in red. **b**–**d** Hierarchically clustered heatmaps for ΔDSS (DSS_Dex_ – DSS_Ctrl_) values for selected kinase inhibitors (**b**), conventional chemotherapy drugs (**c**), and apoptotic modulators (**d**).

We performed DSRT using four OC cell lines (JHOS2, Kuramochi, OVCAR3, and OVCAR3cis) and three PDCs (FMOC04, FMOC09 and FMOC11) followed by unsupervised hierarchical clustering of ΔDSSs (DSS_DEX_ − DSS_Ctr_ for each drug). Altogether, OVCAR3, OVCAR3cis, JHOS2, and FMOC04 showed higher differences in drug responses in the presence of DEX treatment compared to less-responsive Kuramochi cell line and FMOC09 and FMOC11 (Fig. 5a–d and Supplementary Fig. S4). We observed a significant increase in the efficacy of several kinase inhibitors (Fig. 5b and Supplementary Fig. S5) in the presence of DEX treatment, notably PI3K inhibitors (pictilisib, copanlisib, taselisib, omipalisib, among others), AKT inhibitors (ipatasertib, AZD-5363) and HER/EGFR inhibitors (poziotinib, canertinib, dacomitinib, gefitinib, tesevatinib, erlotinib, among others) in OVCAR3/OVCAR3cis and JHOS2 cell lines, whereas enhanced DEX-mediated drug responses for ipatasertib, dacomitinib, poziotinib, and ravoxertinib were observed in FMOC04. Loss of drug efficacy was noted for AT7519, danusertib, GSK-461364, PF-03758309, BI2536, prexasertib, AZD6738, and alisertib in DEX-treated OVCAR3cis and chemoresistant FMOC04 (Fig. 5b), suggesting common DEX-mediated drug changes in chemoresistant OC models. On the other hand, several chemotherapeutic drugs (Fig. 5c and Supplementary Fig. S5) such as paclitaxel, docetaxel, and gemcitabine lost their efficacy after DEX treatment in OVCAR3, OVCAR3cis, FMOC04, and Kuramochi corroborating with the previous observation^6^. Interestingly, we observed a DEX-mediated decrease of drug efficacy for apoptotic SMAC mimetics AT-406, birinapant as well as NVP-LCL161 in DEX-treated OVCAR3, OVCAR3cis, Kuramochi, JHOS2, FMOC04, and FMOC11 cells (Fig. 5d and Supplementary Fig. S4), suggesting that modulation of apoptotic drug responses could be one mechanism responsible for DEX-mediated drug resistance in OC preclinical models. Interestingly, the mesenchymal or de-differentiated FMOC09 showed a DEX-mediated increase in drug efficacy for RSL3, a ferroptotic inducer, and BRD7116, an inhibitor of leukemic stem cells (Supplementary Fig. S4), suggesting new patient-specific actionable drugs for DEX-treated OC.

### ROR1 targeting increases the efficacy of SMAC mimetics and taxanes drugs in OC

**Fig. 6:**
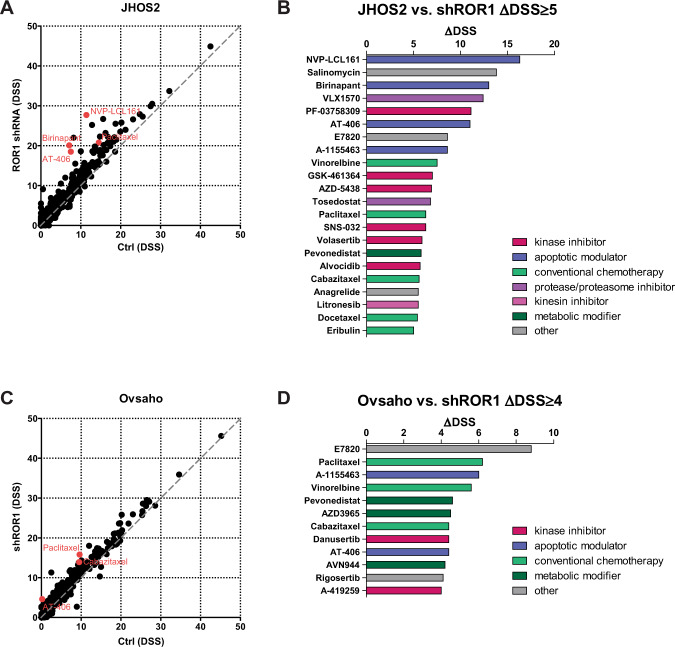
Targeting ROR1 expression enhances the efficacy of SMAC mimetics and taxane agents in JHOS2 and Ovsaho cells. Plot depicting drug-sensitivity scores (DSS) for OC cell line JHOS2 Ctrl vs. ROR1 shRNA (**a**) and Ovsaho Ctrl vs. ROR1 shRNA (**c**). Selected SMAC mimetic drugs and paclitaxel are highlighted in red. Waterfall plot with selected drugs that have ΔDSS (DSS_Dex_ − DSS_Ctrl_) value ≥ 5 for JHOS2 cells (**b**), and ≥4 for Ovsaho cells (**d**).

Since glucocorticoid treatment upregulated ROR1 levels in OC samples, next we investigated changes in drug responses associated with ROR1 targeting in JHOS2 and Ovsaho cells. Analysis of DSSs before and after shRNA ROR1 targeting revealed several drugs that showed enhanced efficacy after doxycycline-induced shRNA ROR1 knockdown (Fig. 6a–d) such as Bcl-x_L_ inhibitor A-1155463, taxane agents (paclitaxel, cabazitaxel), integrin alpha 2 antagonist E7820 as well as anti-apoptotic SMAC mimetics AT-406, birinapant and NVP-LCL161, with DSSs variation for each cell line. Interestingly, AT-406, birinapant, and NVP-LCL161 showed decreased efficacy in DEX-treated JHOS2 in which ROR1 appears to be upregulated (Fig. 2a), indicating that modulation of ROR1 expression could influence the efficacy of SMAC mimetics in OC.

## Discussion

Adjuvant glucocorticoids are widely used in OC clinical treatment to combat the side effects of chemotherapy and to treat symptoms related to advanced cancer. However, numerous studies have indicated that activation of GR signaling via glucocorticoids may spare tumor cells from undergoing apoptosis while impairing the efficacy of chemotherapy^6^. A recent study provided a mechanism by which glucocorticoids may induce metastatic breast cancer and demonstrated that synthetic glucocorticoids such as DEX increased the expression of ROR1, resulting in enhanced metastasis and decreased survival in preclinical models^9^. Previous studies have linked the activation of ROR1 signaling to tumorigenesis, stemness, and drug resistance in OC, and high ROR1 expression was associated with worse OC prognosis^13^.

Here, we tested the effect of the glucocorticoid DEX on ROR1 signaling activation and analyzed the global changes in drug responses in OC preclinical models, including platinum-resistant cells. We developed cisplatin-resistant OVCAR3 cells and observed the upregulation of Wnt5a-ROR2 in OVCAR3cis, consistent with our previous results showing that Wnt5a-ROR2 expression is linked to cisplatin resistance development in OC models^12^. Furthermore, we observed a significant increase in ROR1 protein expression following DEX treatment in OC cells, and this correlated with the upregulation of ROR1 downstream signaling such as RhoA, YAP/TAZ, and BMI-1 levels. DEX-mediated upregulation of ROR1 and its downstream signaling was observed in both conventional cell culture as well as in spheroids. Interestingly, in OC cells cultured in spheroid conditions (both cell lines and PDCs) we detected an increase in ALDH1A1 levels following DEX treatment, strongly suggesting the development of stemness phenotype mediated by this synthetic glucocorticoid. Our results are in corroboration with previous findings showing that modulation of ALDH1A1 expression is more easily detected in spheroid cultures^26^. ROR1-dependent upregulation of RhoA, YAP/TAZ, and BMI-1 was demonstrated by inducible shRNA targeting ROR1 expression in JHOS2 cells, which abrogated DEX-mediated increase in RhoA, YAP/TAZ, and BMI-1 expression (Fig. 3a). Moreover, pharmacological inhibition of YAP/TAZ by verteporfin downregulated ROR1 levels, indicating the existence of a feedback regulatory loop linking YAP/TAZ and ROR1 signaling. Ultimately, Wnt5a-mediated upregulation and nuclear localization of YAP1 was observed in JHOS2 cells, and this effect was ROR1-dependent. Conclusively, our results show that DEX treatment elevated ROR1 expression, which in turn enhanced RhoA, YAP/TAZ, and BMI-1 levels in OC tumor cells and is indicative of a DEX-mediated stemness phenotype via ROR1 signaling. Interestingly, ROR1 downregulation did not affect GR expression, suggesting an indirect modulation between GR signaling activation and ROR1 expression.

The expression of E-cadherin and its associated differentiation markers has relevant biological significance for OC disease outcome^27^. Low E-cadherin levels were associated with advanced OC stages and the development of peritoneal metastasis^21^. Moreover, decreased E-cadherin expression was detected in ascites spheroids compared to matched solid tumors^28,29^ while another study associated decreased E-cadherin but increased N-cadherin expression with a mesenchymal, or de-differentiated, a subtype of HGSOC that is linked with shorter OS compared to other subgroups^23^. In our PDCs collection, we also identified that FMOC09 exhibited a mesenchymal or de-differentiated gene expression signature compared to other PDCs. Moreover, our results showed that DEX could modulate the expression of cadherins and the differentiation mechanism in OC spheroids, although the spectrum of DEX-mediated differentiation outcomes observed here suggests variations in underlying molecular mechanisms, which reflects the complexity of OC subtypes and their gene signature. Nonetheless, our data clearly indicate that glucocorticoids alter the level of differentiation markers in spheroid models and could therefore influence OC disease progression, denoting clinical significance.

We tested the effect of glucocorticoids on 528 oncology drug responses to identify DEX-modulated synergistic or antagonistic effects with translational relevance for OC treatment. As expected, DEX treatment decreased the efficacy of several chemotherapy drugs, most significantly taxanes (paclitaxel, cabazitaxel, and docetaxel) and alkaloid microtubule depolymerizers (vinorelbine, vinblastine, and vincristine). Loss of chemotherapy drugs efficacy in glucocorticoid-treated OC samples corroborated with previous observations, since DEX has been shown to exert a cytoprotective effect when used in combination with standard chemotherapy and to contribute to chemotherapeutic resistance^30,31^. Interestingly, we observed a significant loss of efficacy for SMAC mimetics and IAP antagonists AT-406, birinapant, and NVP-LCL161 in all DEX-treated OC cell lines and FMOC4. AT-406 is a potent monovalent SMAC mimetic that induces rapid degradation of cIAP1 protein and inhibits cancer tumor growth^32^. Birinapant is a bivalent SMAC mimetic that preferentially targets TRAF2-associated cIAP1 and cIAP2 to inhibit TNF-induced NF-κB activation, and has been shown to have antitumor activity in ovarian and colorectal cancers^33^. NVP-LCL161 is a first-in-class oral SMAC mimetic that induces degradation of cIAP1 and has demonstrated single-agent activity in human tumor xenograft models, with basal production of TNF-α and NF-κB inhibition as a common mechanism^34,35^. It is currently unknown how glucocorticoids could decrease the efficacy of SMAC mimetics in OC, although several possible mechanisms could be involved. Modulation of NF-κB activation is one plausible mechanism and previous reports have indicated that glucocorticoids could inhibit NF-κB either indirectly through enhanced transcription of IκBα or directly via competition between coactivator proteins p65 and GR^36–39^. Accordingly, we detected a DEX-dependent upregulation of IκBα in DEX-treated OVCAR3/OVCAR3cis and FMOC04 cell lysates (Fig. S6).

A small phase II clinical trial with birinapant monotherapy for chemoresistant OC patients did not yield significant results^40^, suggesting that combinatorial treatments should be considered. SMAC mimetics were shown to work in synergistic lethality with other drugs such as chemotherapy drugs in solid tumors (paclitaxel, carboplatin, cisplatin, daunorubicin, among others) or with targeted therapies such as TRAIL receptor agonists, epigenetic drugs, or immunotherapies^41^. We detected a strong synergistic lethality between ROR1 targeting and SMAC mimetics AT-406 (in JHOS2 and Ovsaho cells), birinapant, and NVP-LCL161 (in JHOS2 cells), and this combination could be considered for the development of new treatment strategies in chemoresistant OC. ROR1 monoclonal antibody cirmtuzumab is currently in phase I-II clinical trials (NCT02776917) for chemoresistant breast cancer in combination with paclitaxel^19^. Interestingly, we detected the same synergistic effect in JHOS2 and Ovsaho cells with ROR1 targeting and paclitaxel, strongly suggesting that this combinatorial treatment should be tried in OC clinical settings.

On the other hand, increased sensitivities were observed for multiple kinase inhibitors in DEX-treated OC cell lines, as well as for AKT inhibitor ipatasertib and ERK inhibitor ravoxertinib in FMOC04. Ipatasertib sensitivity corroborated our western blot results (Fig. 2b), showing enhanced DEX-mediated AKT phosphorylation in FMOC04. Also, specific DEX-mediated enhanced drug responses for RSL3, a ferroptotic inducer, and BRD7116, an inhibitor of leukemic stem cells were detected in FMOC09, indicative of patient-specific drug responses that could be detected using our ex vivo DSRT platform. Taken together, our DRST screens have identified previously unknown glucocorticoid-mediated drug responses in OC cells, such as DEX-mediated loss of efficacy for SMAC mimetics, which could be reversed by targeting ROR1 expression.

GR is a nuclear hormone receptor activated by endogenous cortisol and synthetic glucocorticoids^42^. Several lines of evidence have involved GR signaling activation in tumorigenesis and cancer progression. For instance, high GR expression that correlates with increased GR activity was associated with a significant decrease in median progression-free survival (PFS) of OC patients^43^. Physiological stress-mediated activation of GR signaling has also been shown to associate with poor patient outcome. Higher levels of stress hormones were found in breast cancer patients with metastatic disease than in age-matched healthy women or patients without metastases^44^ and in other studies, abnormal cortisol rhythms corresponded to shorter survival for patients with advanced breast or OC^45,46^. Our study describes a new molecular mechanism for how GR signaling activation negatively impacts OC disease outcome by promoting ROR1-stemness, differentiation, and drug resistance, highlighting an important therapeutic role for ROR1 in OC.

## Supplementary information

Supplementary Materials and Methods

Figure S1

Figure S2

Figure S3

Figure S4

Figure S5

Figure S6

Table S1

Table S2

Table S3
