# Supplementary material for: Glucocorticoids induce differentiation and chemoresistance in ovarian cancer by promoting ROR1-mediated stemness
Source: Cell Death Dis. 2020 Sep 23;11(9):790. doi: 10.1038/s41419-020-03009-4 (PMC7522257; doi:10.1038/s41419-020-03009-4)
Supplement: Supplementary file 1 — Supplementary Materials and Methods [file 41419_2020_3009_MOESM1_ESM.pdf]

Materials and methods

Cell culture

Ovarian cancer cell lines OVCAR3, Kuramochi and Ovsaho were grown in RPMI 1640 media (Lonza, Basel, Switzerland) supplemented with 10% fetal bovine serum, 2mM L-glutamine, and Primocin™ (InvivoGen, San Diego, CA, USA). JHOS2 cells were grown in DMEM/F-12 media (Gibco™, Thermo Fisher Scientific, Waltham, MA, USA) supplemented with 10% fetal bovine serum, 1x MEM Non-essential amino acid solution, and Primocin™. SKOV3 cells were grown in McCoy's 5A (modified) media (Gibco™, Thermo Fisher Scientific) supplemented with 10% fetal bovine serum and Primocin™. Cell counting was done with Countess or Countess II automated cell counter (Invitrogen™, Thermo Fisher Scientific)

A cisplatin resistant version of OVCAR3 cell line (OVCAR3cis) was generated from the parental OVCAR3 cell line by culturing cells with increasing concentrations of cisplatin. OVCAR3cis maintenance cultures were supplemented with 1µM cisplatin to sustain the cisplatin resistant phenotype.

For ROR1 silencing, stable shRNA cell lines expressing control shRNA and ROR1 shRNA were produced as described previously[1] using inducible pLKO-Tet-On lentiviral expression system[2,3]. Expression of shRNA was induced by 100ng/ml doxycycline (DOX) treatment for at least 2 days and ROR1 silencing was verified by Western blot analysis.

Patient derived cell cultures (PDCs)

All patient material and data were acquired under Institutional Ethical Review Board-approved protocols (No. 56/13/03/03/2014). Informed consent was obtained from all patients. Patient´s clinical characteristics are summarized in Table 1. In order to obtain a sufficient number of PDCs for high-throughput drug testing in a relatively short time we used the protocol described by Liu et al.[4,5] Freshly isolated patients´ cancer cells were cocultured with irradiated fibroblast feeder cells in culture medium supplemented with Rho-kinase (ROCK) inhibitor Y-27632. Ascites fluid from advanced-stage serous ovarian cancer patients was collected from consenting patients and used to initiate primary cultures. Ascites was seeded on regular flasks with MCBM:M105 growth medium supplemented with fetal

bovine serum (10%), 2mM glutamine, and 2mM penicillin/streptomycin. Primary cancer cells were maintained at 37°C with 5% CO2 and passaged weekly. Experiments were performed with early passage cells. PDCs were verified for identical phenocopy with their original tumor samples by next-generation sequencing (data not shown) and the expression of Müllerian marker paired-box 8 (PAX8), a known marker for the diagnosis of epithelial ovarian cancers (Fig. S1A).

Spheroids

For spheroid experiments cells were switched to MBEM media (Misvik Biology, Turku, Finland) supplemented with MBEM supplement mix, 2mM L-glutamine, and Primocin™. Cells were cultured on low attachment plates for 4-6 days. Images were taken with Invitrogen™ EVOS™ FL imaging system (Thermo Fisher Scientific) or Leica DMI LED microscope (Leica microsystems, Wetzlar, Germany) with Nikon DS-fi1 camera (Nikon, Tokyo, Japan).

Cell viability assay

CellTiter-Glo (CTG) 2.0 Assay (Promega, Madison, WI, USA) was used to measure cell viability. Cells were plated to 96- or 384-well plates and treated with indicated drug concentrations. After 72h an equal volume of CTG reagent was added to each well and plate incubated for 30 min at room temperature (RT) before measuring the luminescence with Envision plate reader (Perkin-Elmer, Waltham, MA, USA).

Western blots

Cells were lysed with Triton-X lysis buffer (50 mM Tris-HCl pH 7.5, 10 % glycerol, 150mM NaCl, 1 mM EDTA, 1 % Triton-x-100, 50 mM NaF) supplemented with protease and phosphatase inhibitor cocktails (Bimake, Houston, TX, USA). Protein levels were balanced based on Bradford assay before SDS-PAGE separation and transfer to nitrocellulose membranes. The following primary antibodies were used for blocked membranes: Akt (#2920), phospho-Akt (Ser473) (#4060), ALDH1A1 (#36671), BMI-1 (#6964), Claudin-1 (#13255), E-Cadherin (#3195), Glucocorticoid Receptor NR3C1 (#12041), IκBα (#4814), N-Cadherin (#13116), RhoA (#2117), ROR1 (#16540), ROR2 (#88639), Vimentin (#5741), Wnt5a/b (#2530), YAP/TAZ (#8418) and ZO-1 (#8193) from Cell Signaling Technology (Dancers, MA, USA), and β-

tubulin (#sc-166729) from Santa Cruz Biotechnology (Dallas, TX, USA). Detection was done using IRDye® 800CW Donkey anti-Mouse IgG or IRDye® 680RD Donkey anti-Rabbit IgG (LI-COR, Lincoln, NE, USA) secondary antibodies and blots were scanned with Odyssey® CLx Imaging system (LI-COR). Image analysis was done using the Image Studio Lite software (LI-COR).

Immunofluorescence

Stable transfected JHOS2 shCtrl and JHOS2 shROR1 cells were plated on 24-well plates and treated with doxycycline for 2 days before switching them to starvation media (without FBS). After 24h starvation half of the wells were stimulated with 50ng/ml Wnt5a for 2h. Cell were then fixed with 4% PFA in PBS solution for 15 min at RT. Permeabilization was done with 0.1% Triton X in PBS for 10min at RT followed by blocking with 1% BSA in PBS for 1h at RT. Cells were incubated overnight at 4°C with YAP antibody (#14074, Cell Signaling Technology, 1:100 dilution), followed by secondary antibody, Alexa Fluor Plus 594 donkey anti-rabbit IgG (Invitrogen™, Thermo Fisher Scientific, 1:1000 dilution), incubation for 1h at RT. Cells were counterstained with DAPI. Images taken with Invitrogen™ EVOS™ FL imaging system (Thermo Fisher Scientific).

Gene expression analysis

Total RNA was isolated from PDCs using total RNeasy kit (Qiagen, Germany). Quantity and quality of the RNA samples were assessed by Qubit (Thermo Fisher Scientific, USA) and Bioanalyzer (Agilent Technologies, USA). RNA with an RNA integrity number (RIN) > 8 was used to derive libraries and the sequencing was performed with Illumina HiSeq system (Illumina) as previously described[6]. The expression of Wnt-related genes or differentiation-related markers for ovarian cancer subtype characteristic genes was derived from RNA-seq analysis. Hierarchical clustering analysis was performed using log2 transformation of transcripts per kilobase million (TPM) values derived from RNA-Seq data from samples of five PDCs. Clustering was done separately on genes involved in the Wnt pathway, based on KEGG pathway definitions[7], and on genes associated with mesenchymal, de-differentiated, cell type[8]. In both cases clustering was done with the cluster module of the SciPy library[9], using a Euclidean

distance metric and Ward linkage variance. Visualization of clusters were done with Matplotlib [10] and Seaborn[11]. In presentation of clustering in Wnt pathway genes a divergent expression color panel was selected to highlight absolute expression of genes across and within samples. In the mesenchymal plot values were standardized by row (zero to one) and colored by progression of expression to highlight variation of genes among samples. RNA-seq data for Wnt pathway genes is in Supplementary table 2 and for mesenchymal genes in Supplementary table 3.

Drug sensitivity and resistance testing (DSRT)

DSRT was done as described previously[12,13] for ovarian cancer cell lines and PDCs with and without dexamethasone treatment (100nM) for 72 hours. Briefly, 1 000-1 500 cells were added to wells of 384-well plates with drugs pre-plated over a 10 000-fold concentration range (in five concentrations). After a 3-day incubation at 37 °C, cell viability was measured using the Cell Titer-Glo reagent (Promega) and plates were read with PHERAstar FS (BMG Labtech, Ortenberg, Germany). For each drug dose response curves were generated and DSS were calculated as previously described[12,13]. In short, DSS is a measure of drug-response based on the area under the dose response curve that captures both the potency and the efficacy of the drug effect. It integrates complementary information extracted by half-maximal inhibitory concentration (IC50), slope and minimal and maximum asymptotes. Only the drugs with ΔDSS (DSS_{Dex}-DSS_{Ctrl}) ≥5 or ≤-5 in any of the tested cell lines were analyzed further.

Flow cytometry

ROR1 staining was done with anti-ROR1-PE 2A2 antibody (Miltenyi Biotec, Bergisch Gladbach, Germany) according to manufacturer's instructions. Cell detaching was done with Accutase® cell detachment solution (Innovative Cell Technologies, San Diego, CA, USA) to preserve cell surface ROR1. Flow cytometry samples were run with BD Accuri C6 (BD Biosciences, San Jose, CA, USA) and analyzed with BD Accuri C6 software.

References

1. Karvonen, H. *et al.* Crosstalk between ROR1 and BCR pathways defines novel treatment strategies in mantle cell lymphoma. *Blood Adv* **1**, 2257–2268 (2017).
2. Wiederschain, D. *et al.* Single-vector inducible lentiviral RNAi system for oncology target validation. *Cell Cycle* **8**, 498–504 (2009).
3. Wee, S. *et al.* PTEN-deficient cancers depend on PIK3CB. *Proc. Natl. Acad. Sci. U.S.A.* **105**, 13057–13062 (2008).
4. Liu, X. *et al.* ROCK inhibitor and feeder cells induce the conditional reprogramming of epithelial cells. *Am. J. Pathol.* **180**, 599–607 (2012).
5. Liu, X. *et al.* Conditional reprogramming and long-term expansion of normal and tumor cells from human biospecimens. *Nat Protoc* **12**, 439–451 (2017).
6. Kumar, A. *et al.* The impact of RNA sequence library construction protocols on transcriptomic profiling of leukemia. *BMC Genomics* **18**, 629 (2017).
7. Kanehisa, M., Goto, S., Furumichi, M., Tanabe, M. & Hirakawa, M. KEGG for representation and analysis of molecular networks involving diseases and drugs. *Nucleic Acids Res.* **38**, D355-360 (2010).
8. Tothill, R. W. *et al.* Novel Molecular Subtypes of Serous and Endometrioid Ovarian Cancer Linked to Clinical Outcome. *Clin Cancer Res* **14**, 5198–5208 (2008).
9. Oliphant, T. E. Python for Scientific Computing. *Computing in Science Engineering* **9**, 10–20 (2007).
10. Hunter, J. D. Matplotlib: A 2D Graphics Environment. *Computing in Science Engineering* **9**, 90–95 (2007).
11. Michael Waskom *et al. seaborn: v0.5.0 (November 2014)*. (Zenodo, 2014). doi:10.5281/zenodo.12710.
12. Pemovska, T. *et al.* Individualized Systems Medicine Strategy to Tailor Treatments for Patients with Chemorefractory Acute Myeloid Leukemia. *Cancer Discov* **3**, 1416–1429 (2013).

13. Yadav, B. *et al.* Quantitative scoring of differential drug sensitivity for individually optimized anticancer therapies. *Sci Rep* **4**, 5193 (2014).
