# Supplementary figures and images for: Glucocorticoids induce differentiation and chemoresistance in ovarian cancer by promoting ROR1-mediated stemness
Source: Cell Death Dis. 2020 Sep 23;11(9):790. doi: 10.1038/s41419-020-03009-4 (PMC7522257; doi:10.1038/s41419-020-03009-4)

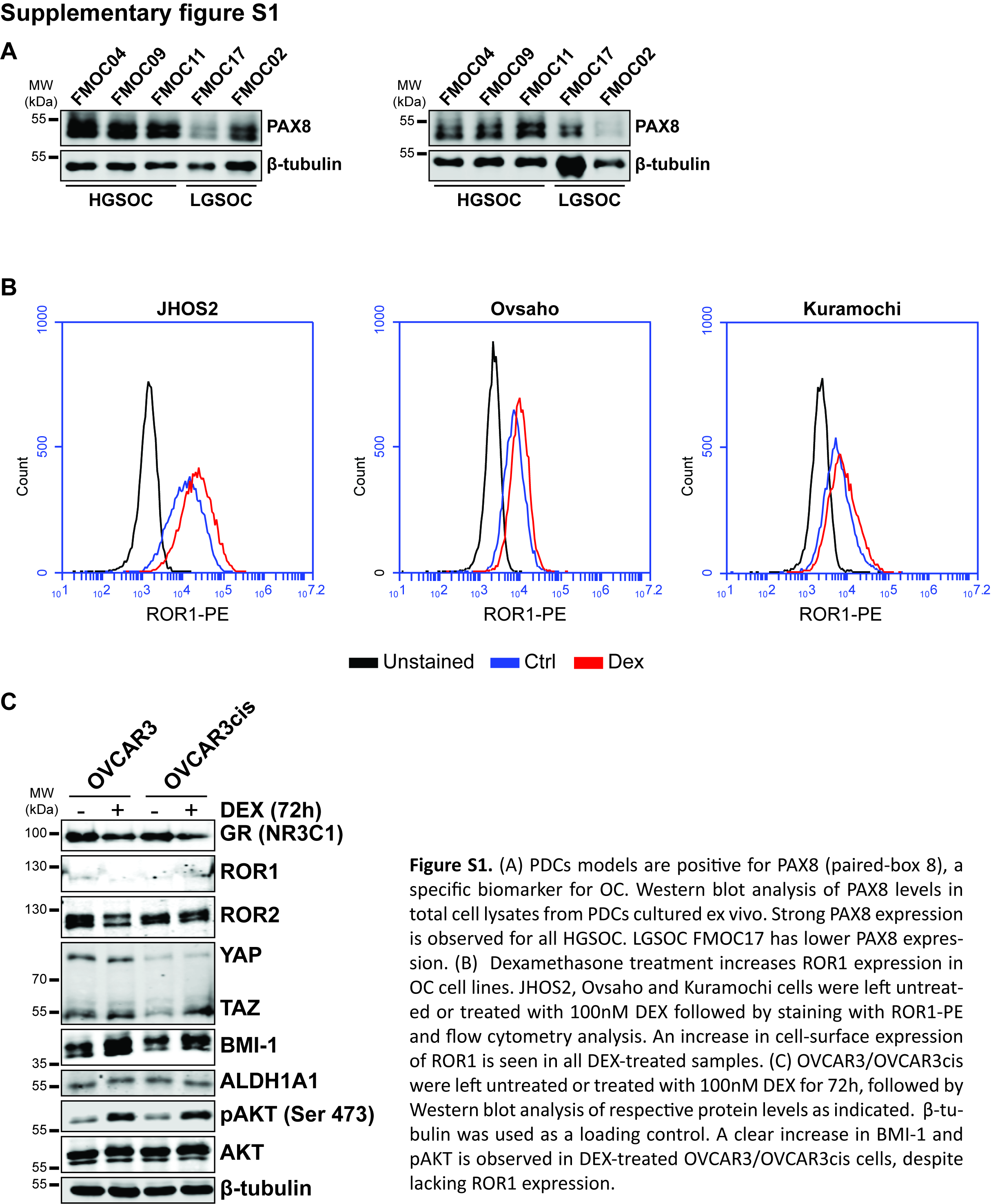

Supplement: Supplementary file 2 — Figure S1 [file 41419_2020_3009_MOESM2_ESM.tif]

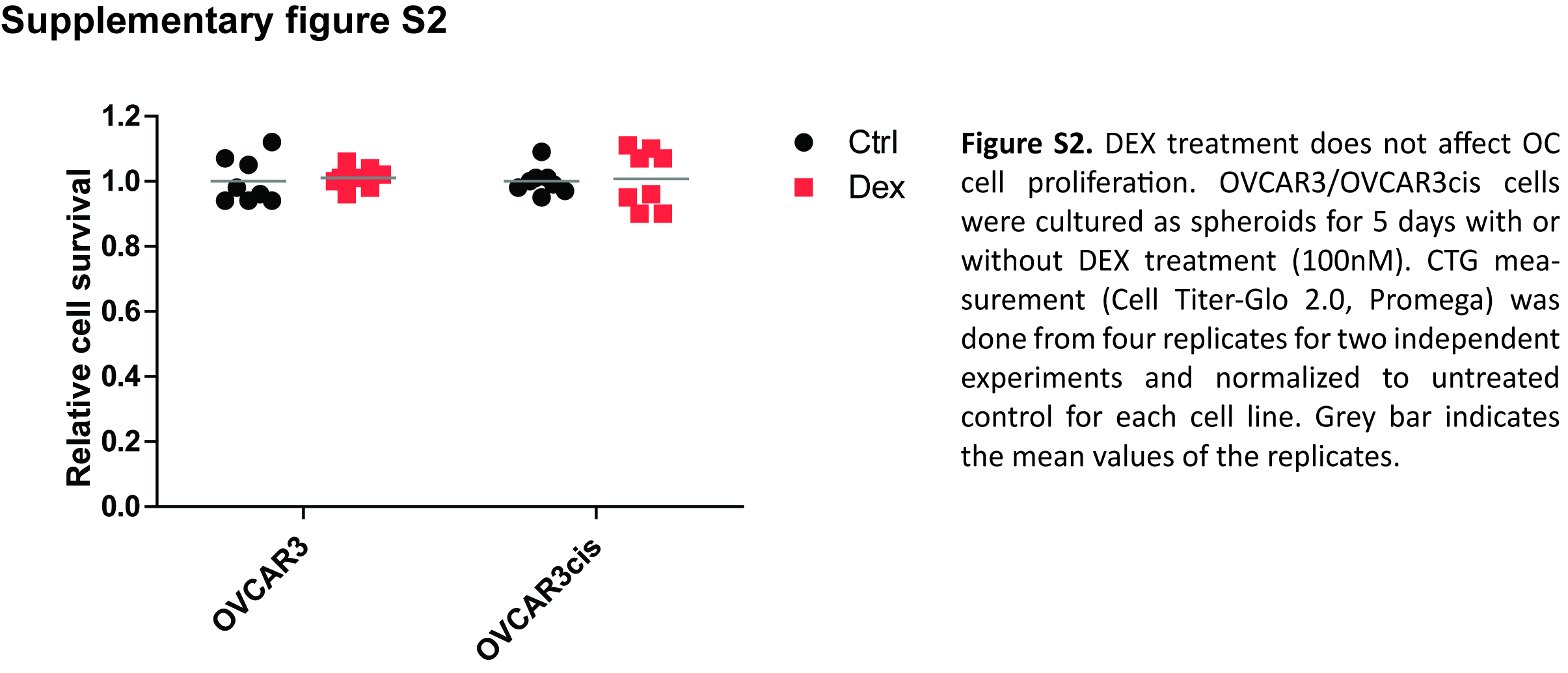

Supplement: Supplementary file 3 — Figure S2 [file 41419_2020_3009_MOESM3_ESM.tif]

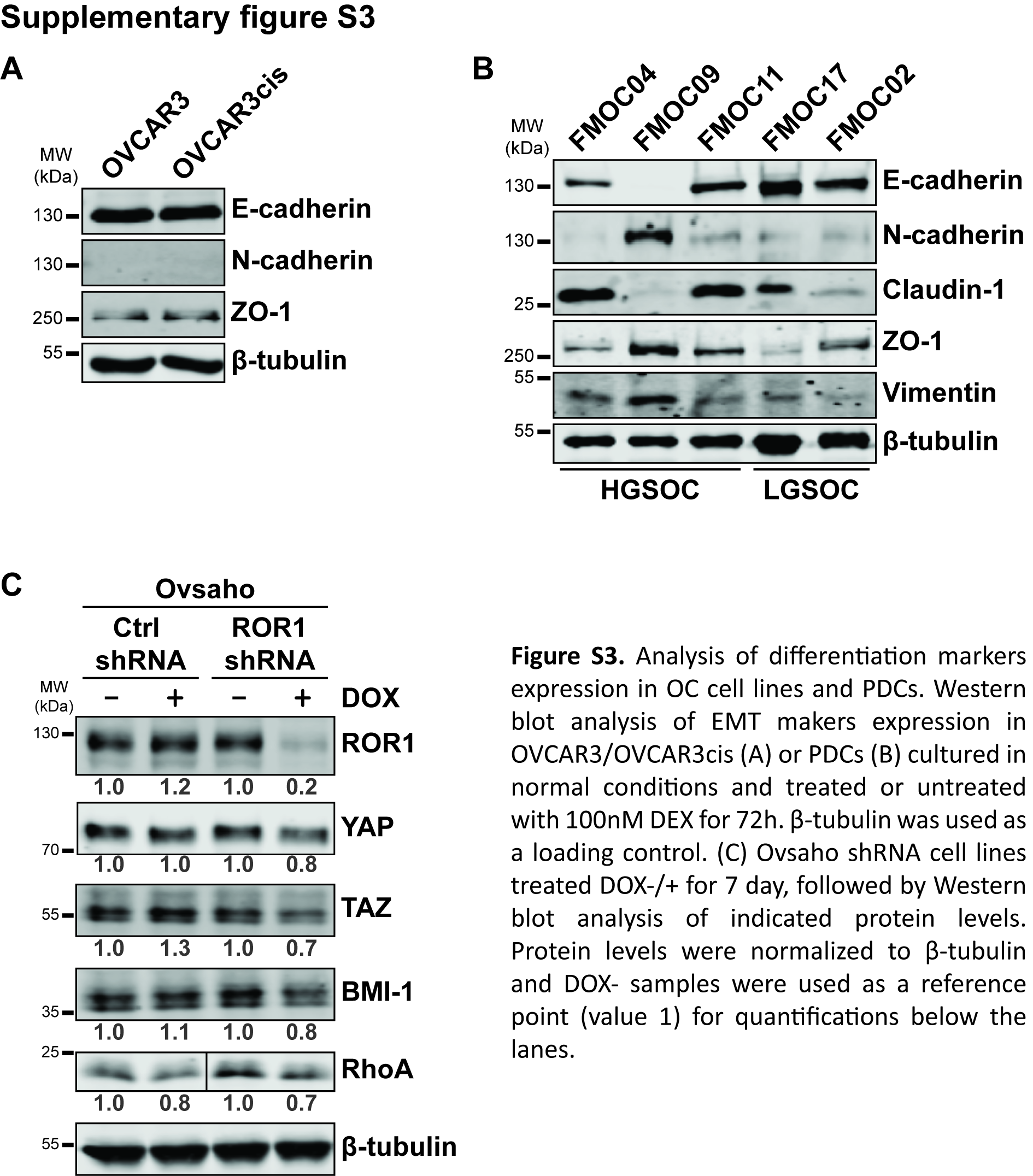

Supplement: Supplementary file 4 — Figure S3 [file 41419_2020_3009_MOESM4_ESM.tif]

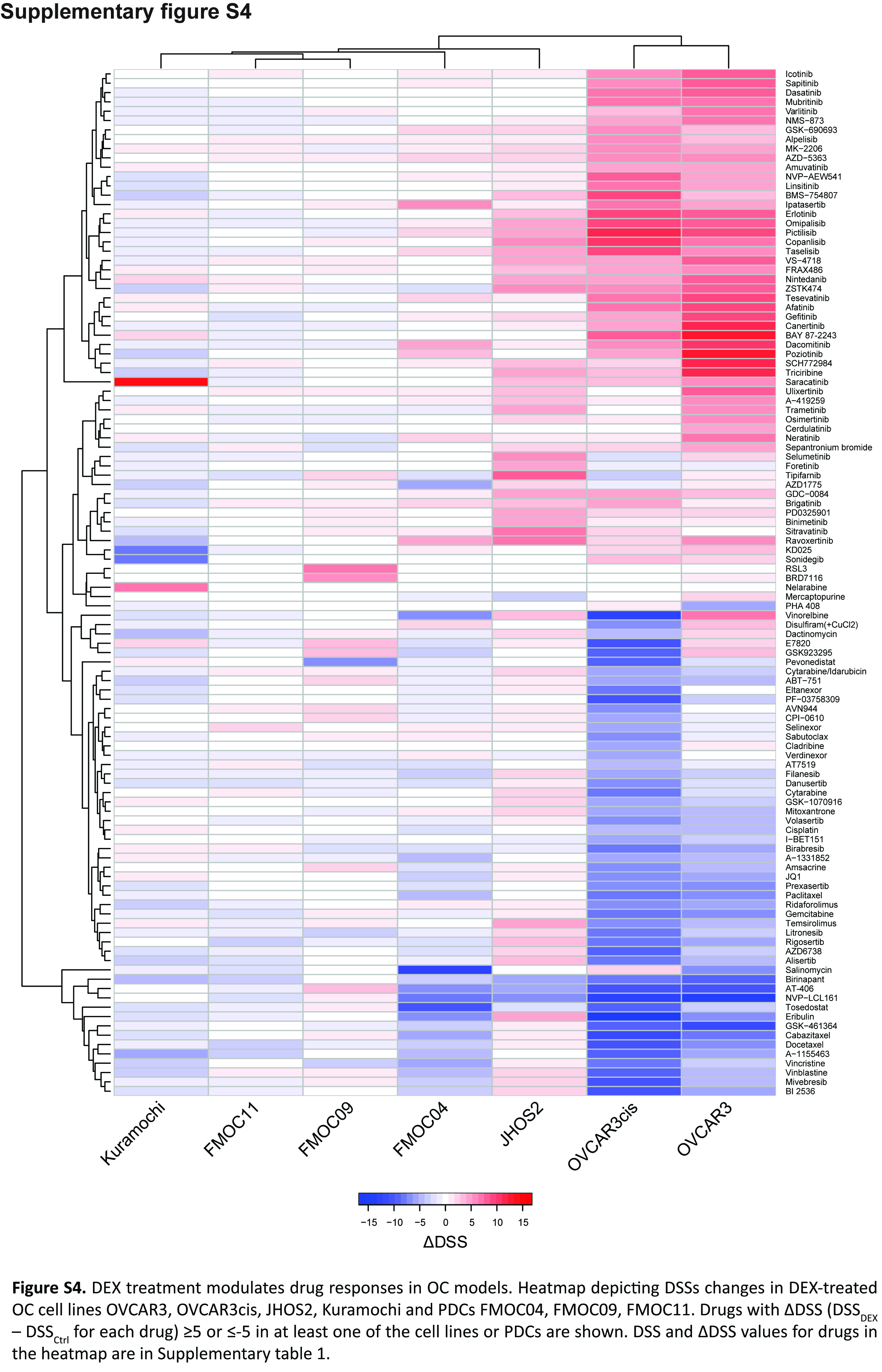

Supplement: Supplementary file 5 — Figure S4 [file 41419_2020_3009_MOESM5_ESM.tif]

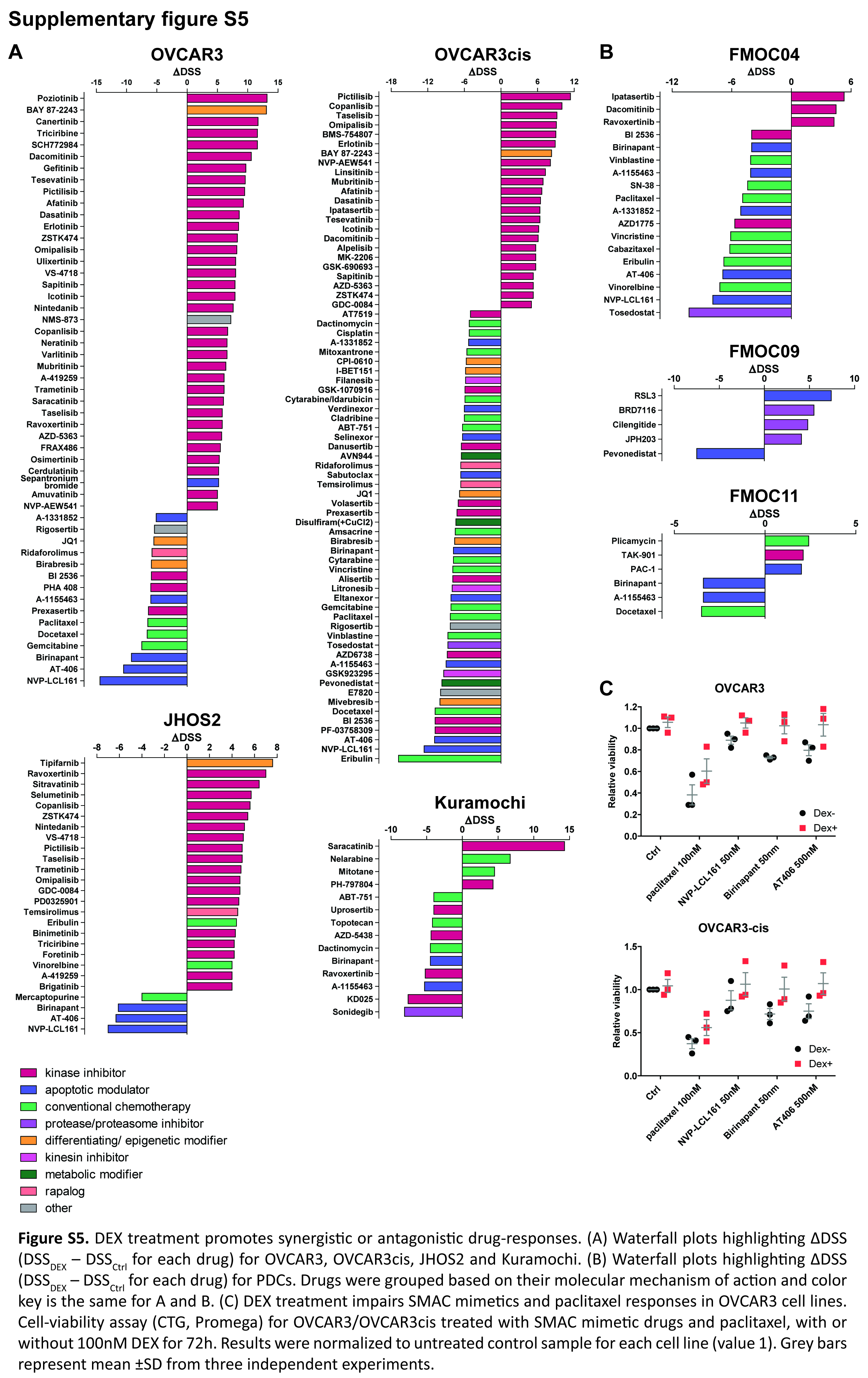

Supplement: Supplementary file 6 — Figure S5 [file 41419_2020_3009_MOESM6_ESM.tif]

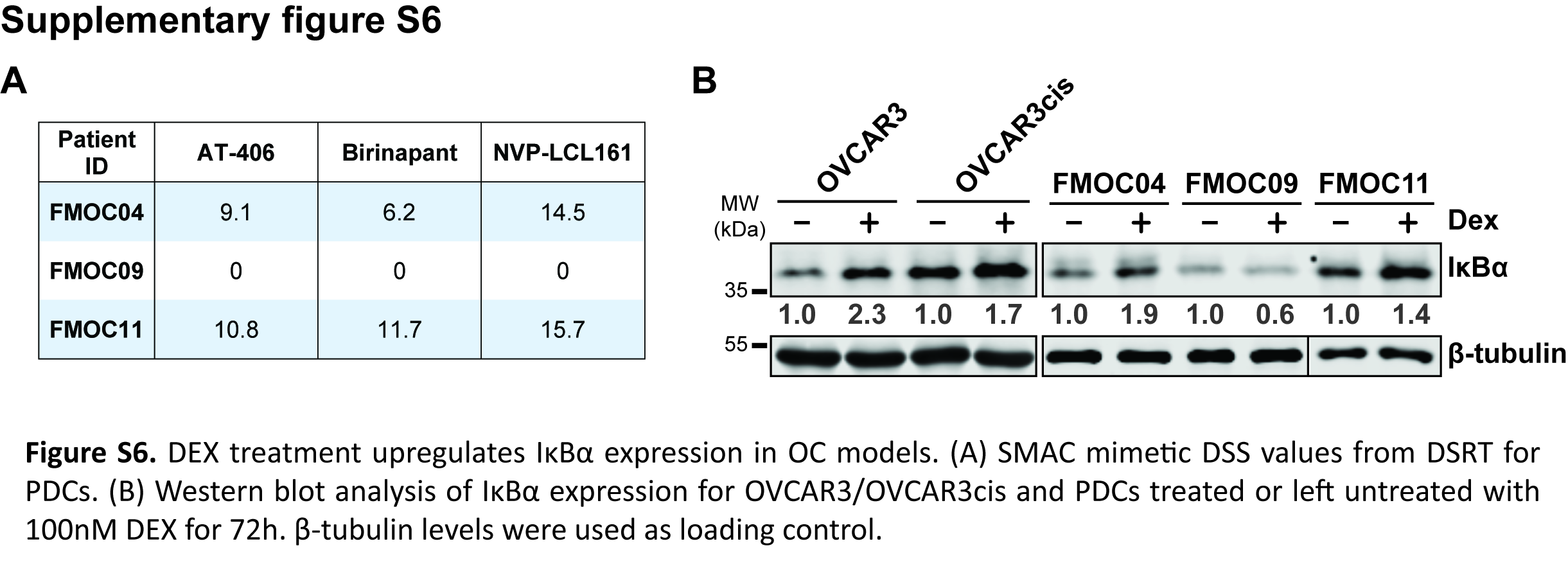

Supplement: Supplementary file 7 — Figure S6 [file 41419_2020_3009_MOESM7_ESM.tif]
